# A Medico-Legal Manual

**Published:** 1901-10

**Authors:** 


A Medico-Legal Manual. By William W. Keysor, Lecturer on Medical Jurisprudence in the Omaha Medical College and Judge of the District Court, Omaha, Nebraska. Duodecimo, pp. 316. Omaha: Burkley Printing Co. 1901. [Price, $2.00.]

The work before us aims to present and emphasise the legal side of medical jurisprudence, to furnish a variety of useful information on legal topics of particular importance to medical practitioners, and to familiarize the doctor with those legal terms and principles which he ought to know and comprehend in order to acquit himself creditably as an expert witness. It is a work alike useful for dentists, and pharmacists, as well as physicians and surgeons, because it contains matter of vital importance to the healing profession and its allies in regard to contracts for professional services, witness fees, evidence, neglience, malpractice suits and other information of interest to the profession.

It is clear and concise, exceedingly well written, and touches the vital points of all questions discussed. Some few excusable errors have crept into the work. For instance, on page 185, in giving the average weight and length of still-born children, the author employs the word "infant," instead of "fetus." The chapters on Life insurance, Identification of persons, Persons found dead, Fruits of cohabitation, contain valuable legal information and make exceedingly interesting reading.

This work is to be commended to all the learned profession.

